# An Improved Robust ESKF Fusion Positioning Method with a Novel UWB-VIO Initialization

**DOI:** 10.3390/s26061804

**Published:** 2026-03-12

**Authors:** Changqiang Wang, Biao Li, Yuzuo Duan, Xin Sui, Zhengxu Shi, Song Gao, Zhe Zhang, Ji Chen

**Affiliations:** 1 School of Geomatics, Liaoning Technical University, Fuxin 123000, China; wangchangqiang@lntu.edu.cn (C.W.); 2304070102@stu.lntu.edu.cn (Y.D.); suixin@lntu.edu.cn (X.S.); 47211047@stu.lntu.edu.cn (Z.S.); 47221105@stu.lntu.edu.cn (S.G.); 472320856@stu.lntu.edu.cn (Z.Z.); 4724200837@stu.lntu.edu.cn (J.C.); 2 The Ordos Institute, Liaoning Technical University, Ordos 017000, China

**Keywords:** mobile robot, visual inertial odometer, ultra-wideband, initialization, robust ESKF, fusion positioning

## Abstract

**Highlights:**

**What are the main findings?**

- A direction-consistent constrained initialization model is proposed to jointly optimize scale and heading, achieving consistent alignment between VIO and UWB coordinate frames without external calibration.
- An improved residual-weighted robust ESKF fusion method adaptively suppresses UWB multipath and NLOS-induced outliers, effectively reducing VIO drift and enhancing localization robustness.

**What are the implications of the main findings?**

- The proposed UWB–VIO framework enables high-precision and stable localization for mobile robots in complex indoor environments with illumination variations and feature sparsity.
- The findings provide a practical and robust localization solution for autonomous navigation and mapping in GNSS-denied scenarios.

**Abstract:**

Visual–inertial odometry (VIO) often struggles with illumination variations, sparse visual features, and inertial drift in complex indoor settings, leading to scale uncertainties and accumulated errors. To address these issues, this paper proposes a new UWB–VIO initialization method combined with an enhanced Robust error-state Kalman filter (Robust ESKF) fusion technique for mobile robot localization. During initialization, common problems include scale drift and heading inconsistency. To solve these, a direction-consistent constrained initialization model is developed. By jointly optimizing the scale factor and yaw angle, this model ensures consistent alignment between the visual–inertial and ultra-wideband (UWB) coordinate frames. This approach removes the need for external calibration and independent coordinate transformation, which are typically required by traditional methods. In the fusion process, an improved residual-weighted robust filtering mechanism is employed to minimize the impact of abnormal UWB ranging data and noise interference. This mechanism adaptively suppresses outliers caused by UWB multipath reflections and non-line-of-sight (NLOS) propagation, thereby reducing VIO drift and improving the overall robustness and stability of the localization system. Experiments conducted in narrow-corridor environments, where both UWB and visual sensors are affected by interference, demonstrate that the proposed method significantly reduces trajectory drift and attitude jumps, resulting in better positioning accuracy and trajectory continuity. Compared to conventional UWB–VIO fusion algorithms, the proposed method enhances average localization accuracy by over 50% and maintains stable estimation even in severe multipath interference conditions, demonstrating high precision and strong robustness.

## 1. Introduction

In recent years, mobile robots have been increasingly deployed in various domains such as intelligent manufacturing, unmanned delivery, security inspection, and service robotics, placing ever higher demands on their autonomous localization and navigation accuracy [1,2,3]. Autonomous navigation and localization of mobile robots in complex mixed indoor–outdoor environments have become one of the key research directions in the field of intelligent robotics. The accuracy and stability of localization systems directly affect the performance of core robotic functionalities, including perception, path planning, and control [4,5]. Reliable and precise positioning is therefore a fundamental prerequisite for achieving fully autonomous navigation. Among existing localization approaches, visual–inertial odometry (VIO) has been widely adopted in autonomous mobile platforms owing to its compact structure, low cost, and high estimation accuracy [6]. By tightly fusing visual and inertial measurements, VIO provides high-frequency motion state estimation. However, its performance can deteriorate significantly under illumination variations, sparse visual features, and inertial drift, leading to scale uncertainty and cumulative error growth. Consequently, maintaining global consistency over long-duration or large-scale operations remains a major challenge for VIO-based localization systems.

To reduce the drift inherent in VIO, researchers commonly incorporate external global positioning sources to improve localization accuracy through sensor fusion. Commonly used global localization methods include the Global Navigation Satellite System (GNSS) [7,8,9,10], Ultra-Wideband (UWB) [11], Light Detection and Ranging (LiDAR) [12,13,14], and WiFi-based positioning [15]. Among these, GNSS provides absolute position data in outdoor environments; however, it experiences significant signal degradation or becomes completely unavailable indoors, in underground parking structures, and in urban canyon environments [16]. LiDAR-based localization can deliver high-precision mapping and positioning by utilizing environmental geometric features, but it remains expensive, power-hungry, and highly sensitive to dynamic environmental changes [17,18,19]. Conversely, UWB positioning technology offers high temporal resolution, low power consumption, and strong resistance to multipath interference, enabling stable global constraints even in complex indoor settings. Due to these benefits, UWB–VIO fusion localization has become a key research area, offering a promising solution for achieving accurate and reliable positioning in challenging indoor scenarios [20,21].

Existing research on UWB–VIO fusion can generally be categorized into three types. The first category comprises loosely coupled methods, where UWB positions and VIO trajectories are independently estimated and subsequently aligned and corrected at the coordinate level [22]. The second category includes tightly coupled approaches, in which UWB ranging equations are directly incorporated into the filtering or optimization framework, enabling joint estimation of UWB measurements and VIO states within a unified model [23,24,25]. The third category consists of deep learning or graph-optimization-based joint modeling methods, which achieve nonlinear fusion by learning scale factors or estimating alignment parameters between the two modalities [26].

Although existing methods have made significant progress in accuracy and robustness, most fusion approaches still rely on independent coordinate transformations and initialization procedures. Since the VIO coordinate frame is defined within a local inertial reference system, while UWB anchor positions are expressed in a global world coordinate frame, scale and heading inconsistencies naturally occur between the two [27]. Traditional methods typically rely on offline calibration or external measurements to estimate transformation parameters. However, such processes not only raise system deployment costs but also risk introducing substantial initialization errors and secondary error propagation in dynamic environments. Therefore, UWB-VIO initialization remains a key challenge for effective UWB-VIO fusion localization. The core of UWB-VIO initialization involves directly aligning the two coordinate systems and establishing their scale relationship at the algorithmic level [28].

To address this, numerous studies have proposed initialization strategies integrated into the fusion process, aiming to achieve coordinate alignment through scale estimation, directional constraints, or multi-anchor geometric information [29,30,31]. Existing approaches can generally be classified into three categories. The first introduces UWB ranging measurements into the VIO backend optimization to enable adaptive estimation of the scale factor. A representative method directly incorporates UWB ranging residuals into the nonlinear optimization framework of VIO, allowing iterative refinement of both scale parameters and camera trajectories within a unified optimization space [29]. The second category exploits UWB directional constraints—such as angle of arrival and time of flight—to establish joint constraint models, enabling real-time correction of heading errors during the fusion process [30]. The third category leverages multi-anchor geometric constraints, jointly optimizing rotation and scale by minimizing distance residuals, thereby achieving coordinate unification without the need for offline calibration [31]. At present, research on UWB–VIO fusion initialization is evolving from traditional offline calibration schemes toward online, real-time multi-parameter joint estimation frameworks. Nevertheless, several critical challenges remain: how to realize real-time integrated estimation of scale, heading, and coordinate transformation under dynamic conditions; how to ensure stable acquisition of UWB ranging data in the presence of multipath interference and signal instability; and how to maintain VIO observability in feature-degraded environments [32,33].

To address the aforementioned challenges, this paper proposes a novel high-precision localization method that integrates a new UWB–VIO initialization scheme with an improved Robust Error-State Kalman Filter (Robust ESKF). To mitigate scale drift and heading inconsistency during initialization, the scale factor and yaw angle are introduced as joint optimization variables. A novel multi-anchor direction-consistency-constrained model is then established to jointly optimize VIO and UWB, thereby achieving coordinate system unification at the algorithmic level. This approach not only satisfies real-time computational requirements but also enhances initialization accuracy and stability in dynamic environments with multipath interference. Furthermore, to address the susceptibility of UWB measurements to anomalous ranging and noise disturbances during the fusion process, an improved residual-adaptive weighted robust filtering strategy is developed. This method effectively suppresses abnormal observations caused by multipath effects and non-line-of-sight (NLOS) propagation in UWB ranging, thereby achieving high-precision and robust pose estimation.

This study not only proposes a theoretical initialization mechanism based on multi-anchor direction-consistency constraints, effectively addressing the coordinate-system unification challenge between UWB and VIO, but also enhances system robustness and accuracy through UWB–VIO integration using an improved Robust ESKF fusion framework. The main contributions of this paper are summarized as follows:

1. A UWB–VIO joint initialization method based on multi-anchor direction-consistency constraints is proposed. This method simultaneously estimates the scale factor and yaw angle within a unified optimization framework, thereby eliminating the dependence on external coordinate alignment procedures required by traditional approaches.
2. An improved residual-weighted Robust ESKF fusion framework is developed, which dynamically adjusts the observation noise covariance during the filtering update stage to enhance system robustness in complex indoor environments.
3. A field-validated robotic experimental platform is designed and implemented. Multiple comparative experiments were conducted in narrow-corridor environments where both UWB and visual sensors were subject to interference. The results demonstrate that the proposed method significantly outperforms existing approaches across root-mean-square position error, mean error, and maximum error.

## 2. Framework of UWB-Assisted VIO Initialization and Improved Robust ESKF-Based Fusion Localization System

**Figure 1:**
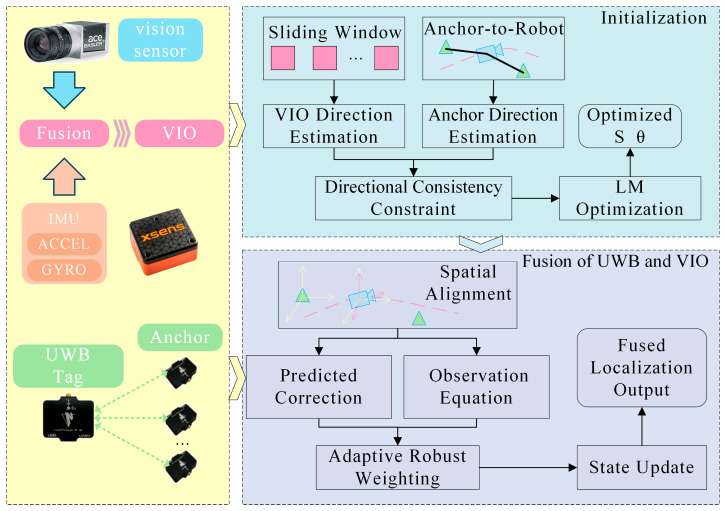
System Framework Flowchart.

The proposed system consists of two primary components: a UWB–VIO joint initialization module and an improved Robust ESKF fusion module. The overall workflow is illustrated in Figure 1. First, preliminary visual–inertial estimates are obtained from the camera and Inertial Measurement Unit (IMU) data, where a sliding-window mechanism is employed to extract a smoothed trajectory direction. Next, UWB directional vectors are derived to establish a direction-consistency constraint between the UWB and VIO systems. Through joint optimization, both the scale factor and yaw angle are estimated simultaneously, enabling high-precision joint initialization and unifying the coordinate systems between UWB and VIO. This initialization strategy effectively mitigates cumulative drift in VIO scale and heading errors, thereby providing stable, consistent initial conditions for subsequent fusion.

After completing the initialization with unified scale and heading, the system proceeds to the fusion stage. In this phase, an improved Robust ESKF framework is employed to fuse UWB and VIO measurements for the dynamic estimation of position and velocity. During the filtering process, an improved residual-weighted Robust ESKF is introduced to suppress abnormal ranging effects caused by UWB signal attenuation and multipath reflections. This approach significantly enhances the accuracy of VIO state updates, effectively mitigating VIO drift while improving robustness, global consistency, and smoother dynamic response performance in complex environments. Moreover, the proposed fusion strategy not only substantially improves positioning accuracy and stability under challenging conditions, but also demonstrates excellent real-time performance and scalability, making it well-suited for high-reliability localization in autonomous mobile robot platforms operating within mixed indoor–outdoor scenarios.

## 3. Joint Estimation of Directional Consistency for UWB–VIO Initialization

**Figure 2:**
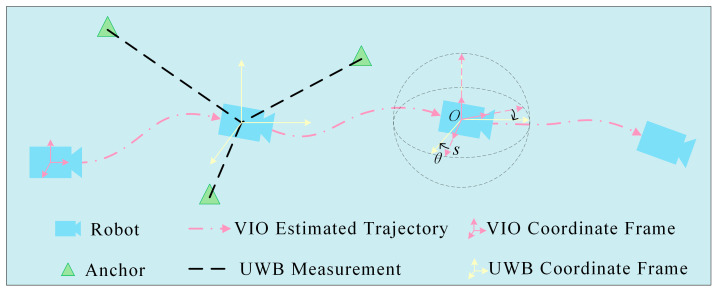
Schematic Diagram of Multi-Anchor Direction Consistency Joint Estimation.

To achieve high-precision spatial alignment between the coordinate systems of the VIO and UWB ranging systems during initialization, this paper proposes an improved UWB–VIO joint initialization method based on multi-anchor directional consistency constraints, as illustrated in Figure 2. In this approach, a sliding-window mechanism is employed to smooth the local trajectory directions of the VIO, thereby establishing directional consistency constraints between VIO and UWB measurements. By incorporating structural consistency and confidence modeling across multiple anchors, the method enables the joint inverse estimation of initialization parameters such as the scale factor and yaw angle. Essentially, this approach fully exploits the global geometric constraints provided by UWB ranging to effectively mitigate scale drift and directional bias commonly encountered during pure VIO initialization, and to eliminate the need to preprocess initialization parameters in UWB–VIO fusion. Consequently, it provides accurate and consistent priors for subsequent fusion-based localization.

### 3.1. Sliding-Window Trajectory Direction Extraction

In VIO systems, visual feature matching and inertial integration are highly susceptible to transient noise, illumination variations, and motion blur, resulting in random fluctuations in the estimated trajectory direction between consecutive frames [34]. Directly employing raw VIO outputs for UWB-assisted initialization may introduce directional inconsistencies, thereby degrading the accuracy and stability of yaw estimation. To reduce such local directional noise, a sliding-window mechanism is introduced to estimate local trajectory direction, producing temporally smooth, geometrically consistent motion direction vectors. Let the three-dimensional position output of the VIO system over time be denoted as $p_{i}$. At time step k, a sliding window of length L is defined as $W_{k}=\{p_{k-L},p_{k-L+1},\ldots ,p_{k}\}$. Within the unit direction vectors between consecutive frames are computed from the continuous trajectory and averaged to obtain the local principal direction:

(1) $${\dot{p}}_{k}=\frac{1}{L}\sum_{i=k-L}^{k-1}\frac{p_{i+1}-p_{i}}{∥p_{i+1}-p_{i}∥}$$

This method effectively suppresses high-frequency noise components induced by visual errors and inertial drift, while preserving the local geometric characteristics of the VIO trajectory.

Since the directional distribution of UWB anchors represents an absolute geometric structure defined in the world coordinate system, whereas the VIO forward direction is defined in the local IMU coordinate frame [35], the yaw angle determines the relative rotation between these two coordinate systems on the horizontal plane. Therefore, when more than two anchors are available, their distribution is non-symmetric, and the robot undergoes motion with trajectory variation, the yaw angle becomes geometrically deterministic and thus observable [36]. Once the robot’s motion direction is obtained from VIO at each time step, the UWB ranging direction vector is computed. The optimal yaw angle can then be derived by minimizing the discrepancy between the VIO motion direction and the UWB ranging direction.

Given the anchor positions and the estimated VIO trajectory, the unit direction vector from an anchor to the tag position can be expressed as:

(2) $$d_{i}^{\left(j\right)}=\frac{p_{A}^{\left(j\right)}-sR_{z}(\theta ){\hat{p}}_{i}}{∥p_{A}^{\left(j\right)}-sR_{z}(\theta ){\hat{p}}_{i}∥}$$

where s denotes the scale factor, $R_{z}(\theta )$ represents the rotation matrix corresponding to the yaw angle θ, and $p_{A}^{\left(j\right)}$ denotes the position of the $j^{\mathrm{th}}$ UWB anchor. The vector $d_{i}^{\left(j\right)}$ represents the unit direction vector pointing from the $j^{\mathrm{th}}$ UWB anchor toward the robot at the frame i.

It should be noted that UWB anchor geometry influences ranging observability and initialization stability. In cases of sparse or suboptimal anchor placement, geometric dilution of precision may increase. However, since the proposed initialization relies on temporal directional consistency over a sliding window rather than instantaneous triangulation, sensitivity to local geometric degeneration is reduced. Furthermore, the robust fusion mechanism prevents filter divergence by adaptively down-weighting unreliable UWB observations, allowing the system to degrade gracefully toward VIO-dominant estimation when necessary. Due to UWB ranging accuracy’s susceptibility to signal attenuation and multipath effects, the spatial distribution of UWB ranging direction vectors may exhibit random deviations and instability, which can interfere with the joint estimation of scale and yaw. To suppress the adverse effects of abnormal ranging values caused by UWB signal attenuation or multipath reflections, confidence weights are constructed based on the received UWB signal strength. This weighting strategy ensures that the UWB ranging direction vectors remain statistically stable and reliable. The confidence weights are defined as follows:

(3) $$w_{i}^{\left(j\right)}=\exp(-(RSS_{max}-RSS_{i}^{\left(j\right)}))$$

where $RSS_{max}$ denotes the maximum received signal strength.

### 3.2. Multi-Anchor Direction Consistency Constraint

After obtaining the smoothed VIO trajectory direction and the UWB direction via a sliding window, the global alignment between the two coordinate systems is achieved by minimizing the angular discrepancy between the VIO and UWB direction vectors. From a geometric perspective, when the yaw is accurately estimated, the VIO trajectory direction—after being rotated into the UWB coordinate frame—should align with the distribution of ranging directions from multiple anchors, thereby achieving unification of the two coordinate systems [37]. To further enhance global observability of yaw and scale, a global kernel matrix is constructed to enforce consistency between the trajectory direction and the anchor-point geometric structure, thereby improving the stability and accuracy of yaw and scale estimation.

Assuming that at least two UWB anchors are utilized during the initialization phase, when the anchors are spatially well-distributed, the joint optimization can be formulated as a least-squares problem with global geometric constraints in the yaw dimension, thereby avoiding the degeneration issue associated with single-anchor configurations. To simplify computation, a structural consistency kernel matrix is introduced to characterize the geometric constraint strength imposed by the anchor orientations on the estimation of yaw and scale.

The covariance tensor of the VIO direction is defined as:

(4) $$Q_{vio}=\sum_{i=1}^{N}{\dot{p}}_{i}{\left({\dot{p}}_{i}\right)}^{T}$$

This tensor characterizes the distribution of the dominant motion directions within the sliding window, along with their correlations, providing a statistical representation of the local trajectory orientation. Geometrically, the principal eigenvector of $Q_{vio}$ represents the dominant motion trend of the VIO trajectory, while the eigenvalue distribution reflects the degree of directional dispersion.

The directional tensor kernel matrix corresponding to each UWB anchor is defined as:

(5) $$K_{uwb}=\sum_{j=1}^{M}\sum_{i=1}^{N}w_{i}^{\left(j\right)}d_{i}^{\left(j\right)}{\left(d_{i}^{\left(j\right)}\right)}^{T}$$

This tensor captures the overall spatial distribution and geometric consistency of the UWB ranging directions, providing a stable global reference. To simplify the computation, this study achieves global alignment between the VIO and UWB directional structures in a statistical sense by minimizing the Frobenius norm of the difference between their directional tensors. The corresponding optimization objective can be expressed as:

(6) $$L(s,\theta )=∥\begin{array}{c} K_{uwb}-sR_{z}^{T}(\theta )Q_{vio}R_{z}(\theta ) \end{array}{∥}_{F}^{2}$$

Expansion of the Frobenius norm:

(7) $$\begin{array}{cc} L(s,\theta ) & =∥K_{uwb}{∥}_{F}^{2}+s^{2}∥Q_{vio}{∥}_{F}^{2}\\ & -2s\mathrm{Tr}\left(K_{uwb}^{T}R_{z}^{T}(\theta )Q_{vio}R_{z}(\theta )\right) \end{array}$$

With respect to the scale s, the function is concave and quadratic; thus, for any given θ, the optimal solution $s^{*}$ is:

(8) $$s^{*}(\theta )=\frac{\mathrm{Tr}(K_{uwb}^{T}R_{z}^{T}(\theta )Q_{vio}R_{z}(\theta ))}{∥Q_{vio}{∥}_{F}^{2}}$$

Substituting Equation (8) into Equation (7) yields:

(9) $$L^{*}(\theta )=∥K_{uwb}{∥}_{F}^{2}-\frac{{\mathrm{Tr}}^{2}(K_{uwb}^{T}R_{z}^{T}(\theta )Q_{vio}R_{z}(\theta ))}{∥Q_{vio}{∥}_{F}^{2}}$$

This step decouples the scale and rotation optimization variables, transforming the problem into a single-variable nonlinear optimization. Furthermore, minimizing $L^{*}(\theta )$ is equivalent to maximizing the trace term $\mathrm{Tr}(K_{uwb}^{T}R_{z}^{T}(\theta )Q_{vio}R_{z}(\theta ))$. The remaining nonlinear least-squares problem is solved using the Ceres Solver [38,39]. By leveraging Ceres’ automatic differentiation and the Levenberg–Marquardt trust-region optimization strategy, efficient joint estimation of yaw and scale parameters is achieved. This approach improves convergence speed while maintaining numerical stability, satisfying the real-time and accuracy requirements of initialization.

## 4. Improved Robust ESKF-Based UWB-VIO Fusion Positioning

**Figure 3:**
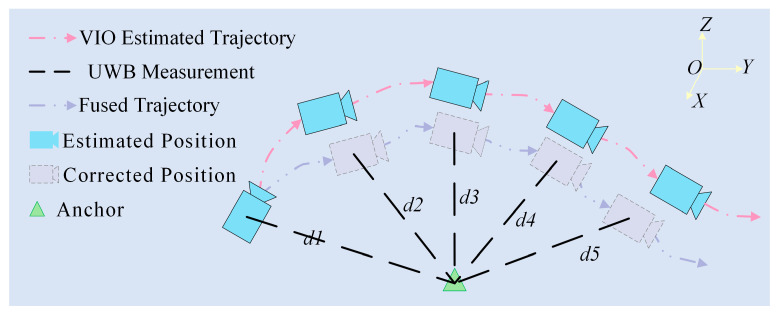
Schematic Diagram of UWB-VIO Fusion Positioning Using the Enhanced Robust ESKF.

Following the UWB-VIO initialization phase, the system obtains an initial state with unified scale and heading, enabling the transition to the dynamic fusion stage for globally robust positioning. An improved residual-weighted Robust ESKF is employed, where residual weighting during the filter update phase mitigates the influence of anomalous observations. The VIO module serves as the reference baseline, while the Kalman filter estimates only the deviations relative to the VIO output. UWB ranging measurements are then used to correct the cumulative drift of VIO, as illustrated in Figure 3. This method adopts an error-state modeling approach, in which the state vector represents the error correction relative to the VIO estimation.

(10) $$\delta X_{k}={\left[\delta x_{k},\delta y_{k},\delta z_{k},\delta v_{x},\delta v_{y},\delta v_{z}\right]}^{T}$$

where $\delta p_{k}={\left[\delta x_{k},\delta y_{k},\delta z_{k}\right]}^{T}$ represents the position error correction relative to the VIO estimation, and $\delta v_{k}={\left[\delta v_{x},\delta v_{y},\delta v_{z}\right]}^{T}$ denotes the velocity error correction.

Unlike the traditional KF, the ESKF does not perform updates directly in the state space. Instead, it linearizes and updates in the error space, followed by error injection to correct the nominal state [40,41,42]. A constant-velocity motion model is adopted for the error correction term, and the discrete-time state transition equation is given by:

(11) $$\delta X_{k|k-1}=F_{k}\delta X_{k-1|k-1}+w_{k},\;w_{k}∼N(0,Q_{k})$$

The state transition matrix is given by:

(12) $$F_{k}=\left[\begin{array}{cccccc} 1 & 0 & 0 & \Delta t & 0 & 0\\ 0 & 1 & 0 & 0 & \Delta t & 0\\ 0 & 0 & 1 & 0 & 0 & \Delta t\\ 0 & 0 & 0 & 1 & 0 & 0\\ 0 & 0 & 0 & 0 & 1 & 0\\ 0 & 0 & 0 & 0 & 0 & 1 \end{array}\right]$$

The error covariance propagation is given by:

(13) $$P_{k|k-1}=F_{k}P_{k-1|k-1}F_{k}^{T}+Q_{k}$$

With the unification of scale and yaw completed during initialization, the transformation from the VIO to the UWB coordinate system can be derived as:

(14) $$p_{k}=S\cdot (R(\theta )\cdot p_{k}^{\mathrm{VIO}})+t$$

The UWB ranging equation can be expressed as:

(15) $$\rho_{i}=h_{i}(X_{k})+v_{i}=∥p_{k}-r_{i}∥+v_{i}$$

where $r_{i}$ denotes the coordinates of the i UWB anchor. During the filter update, linearization is performed at the previous fusion position to ensure the stability of the Jacobian matrix:

(16) $$H_{i,k}=\frac{\partial \rho_{i}}{\partial p_{k}}=\frac{{\left(p_{k-1}^{\mathrm{fusion}}-r_{i}\right)}^{T}}{∥p_{k-1}^{\mathrm{fusion}}-r_{i}∥}$$

To suppress the impact of NLOS and gross ranging errors on filter stability, a residual-based robust weighting function is incorporated into the ESKF framework. First, the innovation vector is formulated, and its residual is computed as follows:

(17) $$\begin{array}{l} z_{i}=\rho_{i}^{\mathrm{VIO}}-\rho_{i}^{\mathrm{UWB}}\\ {\tilde{v}}_{k}=Z_{k}-H_{k}\delta {\hat{X}}_{k|k-1} \end{array}$$

Computation of Residual Covariance:

(18) $$\begin{array}{l} S_{k}=H_{k}P_{k|k-1}H_{k}^{T}+R_{k}\\ s_{i}=\frac{\left|{\tilde{v}}_{i}\right|}{\sqrt{S_{ii}}},\;i=1,2,\ldots ,n \end{array}$$

An improved weighting function is employed to dynamically adjust the measurement noise covariance as ${\tilde{R}}_{i,i}=\omega_{i}^{2}R_{i,i}$ through a robust factor. The robust factor $\omega_{i}$ is defined as:

(19) $$\omega_{i}=\left\{\begin{array}{l} 1,|s_{i}|\leq k_{0}\\ \frac{s_{i}}{k_{0}}\cdot \frac{k_{1}-k_{0}}{k_{1}-s_{i}},k_{0}<|s_{i}|\leq k_{1}\\ 10^{5},|s_{i}|>k_{1} \end{array}\right.$$

where $k_{0}$ and $k_{0}$ are threshold parameters.

The proposed mechanism adaptively adjusts the observation variance to accommodate varying measurement reliability. It automatically suppresses the influence of abnormal UWB ranging observations, thereby improving the system’s robustness and stability under NLOS conditions.

Recalculate the innovation covariance:

(20) $${\tilde{S}}_{k}=H_{k}P_{k|k-1}H_{k}^{T}+{\tilde{R}}_{k}$$

Compute the filter gain and update the state:

(21) $$\begin{array}{l} K_{k}=P_{k|k-1}H_{k}^{T}{\tilde{S}}_{k}^{-1}\\ P_{k|k}=\left(I-K_{k}H_{k}\right)P_{k|k-1}{\left(I-K_{k}H_{k}\right)}^{T}+K_{k}{\tilde{R}}_{k}K_{k}^{T}\\ \delta {\hat{X}}_{k|k}=K_{k}{\tilde{v}}_{k} \end{array}$$

The final fused position is obtained by subtracting the correction estimated by the Kalman filter from the VIO estimate:

(22) $$p_{k}^{\mathrm{fusion}}=p_{k}-\delta {\hat{p}}_{k|k}$$

where $p_{k}$ represents the position estimate obtained after transforming VIO into the UWB coordinate system, and $\delta {\hat{p}}_{k|k}={\left[\delta {\hat{X}}_{k|k}(1),\delta {\hat{X}}_{k|k}(2),\delta {\hat{X}}_{k|k}(3)\right]}^{T}$ denotes the position correction term estimated by the Kalman filter.

For clarity, the experimental execution logic is summarized in Algorithm 1. It describes the initialization stage and the subsequent online fusion procedure implemented on the onboard computing platform.
**Algorithm 1.** Proposed UWB–VIO Fusion Framework**Input:** Visual measurements, IMU measurements, UWB ranging data **Output:** Globally consistent position and orientation estimates //Stage I: UWB–VIO Joint Initialization 1: Acquire visual and IMU data 2: Perform visual–inertial estimation to obtain a preliminary trajectory 3: Apply a sliding-window mechanism to extract the smoothed VIO direction 4: Compute UWB direction vectors 5: Construct direction-consistency constraints between UWB and VIO 6: Jointly optimize scale factor and yaw angle 7: Align UWB and VIO coordinate frames //Stage II: Robust ESKF-Based Fusion 8: Initialize state vector in the ESKF framework 9: while the system is running do 10: Propagate state using IMU measurements 11: Update state using VIO observations 12: Update state using UWB measurements 13: Apply residual-based robust weighting to suppress abnormal UWB measurements 14: Output fused pose and velocity estimates 15: end while

## 5. Experiments and Results Analysis

### 5.1. Experimental Environment and Equipment

**Figure 4:**
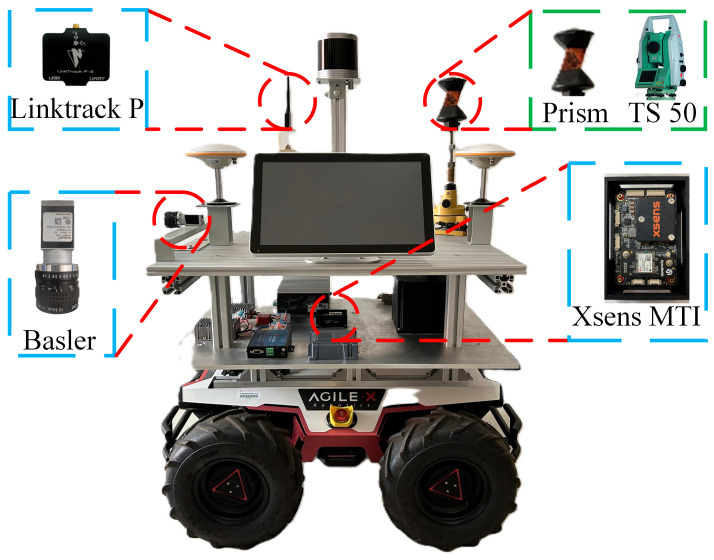
Equipment and Mobile Platform Diagram.

To evaluate the effectiveness and robustness of the proposed positioning method, which combines UWB-assisted VIO initialization with an improved Robust ESKF fusion, field experiments were conducted on an autonomous mobile robot platform. The corresponding experimental setup is illustrated in Figure 4. The robot chassis adopts a differential-drive configuration, and the UWB module utilizes the LinkTrack-P series ultra-wideband positioning system (Nooploop Technology Co., Ltd., Beijing, China). Eight fixed base stations were installed along the corridor of an academic building at a height of approximately 2.0 m, while the mobile unit was mounted on top of the robot. The UWB ranging operates at 5 Hz using the Two-Way Time-of-Flight method. The visual–inertial module consists of a Basler Aca2500-20gc camera (Basler AG, Ahrensburg, Germany) and an Xsens MTI-670 IMU (Xsens Technologies B.V., Enschede, The Netherlands), providing synchronized image and inertial data. The camera captures images at a resolution of 648 × 512 pixels with a frame rate of 10 Hz, and the IMU samples at 200 Hz. A Leica TS50 automatic tracking total (Leica Geosystems AG, Heerbrugg, Switzerland) station was employed to provide the ground-truth reference trajectory for performance evaluation.

**Figure 5:**
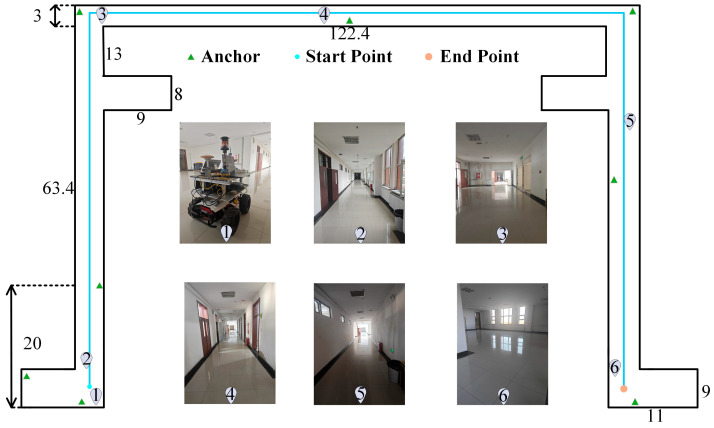
Experimental Setup Diagram. All distances in the figure are expressed in meters (m). Numbers 1–6 indicate the position information corresponding to subfigures (1)–(6).

The experimental environment is shown in Figure 5. The test was conducted in a long corridor of a university teaching building, with a width of only 3 m. The narrow-corridor environment was intentionally selected due to its challenging characteristics for UWB deployment and fusion-based localization. In such constrained spaces, anchor placement flexibility is limited, and multipath interference as well as signal occlusion are more pronounced. Therefore, successful validation in this setting indicates strong robustness of the proposed method. In more open indoor environments, where anchor deployment is less restricted, the system is expected to achieve equal or improved localization performance. It should also be noted that the current framework focuses on planar localization and is not directly designed for multi-floor scenarios.

The UWB anchors were deployed along the corridor, resulting in their nearly collinear alignment with the robot’s direction of motion. This configuration provides limited distance constraints and reduces the system’s observability in the vertical direction. Moreover, wall obstructions and reflective surfaces, such as metal doors and glass windows, attenuate UWB signals and introduce multipath interference, thereby degrading overall fusion accuracy. The long corridor represents a typical visually degraded environment, characterized by sparse feature distribution, highly repetitive structures, and monotonous textures, which significantly reduce the robustness of visual feature extraction and matching. Furthermore, as illustrated in regions 3 and 5 of Figure 5, uneven illumination and local lighting deficiencies inside the corridor reduce the image signal-to-noise ratio, making feature tracking prone to loss and further exacerbating inertial drift and scale uncertainty. In such environments with weak textures, repetitive structures, and low illumination, both the positioning accuracy and long-term stability of VIO systems degrade substantially. To comprehensively evaluate the long-distance positioning accuracy and stability of the proposed fusion method under complex environmental conditions with poor lighting and weak textures, a corridor experiment was conducted. In this experiment, the robot moved from the starting point to the endpoint along the entire corridor, completing a continuous trajectory of approximately 250 m.

During the experiments, the robot was manually driven along a predefined route to maintain approximate trajectory consistency. Each experiment was repeated four times under the same configuration. As the robot was manually operated, minor variations in traversal time inevitably occurred. To better reflect the repeatability of the experiments, the positioning error curves for all individual trials are reported, while the quantitative results correspond to the average error across the repeated experiments.

### 5.2. Experimental Protocol and Results Analysis

Four comparative experiments were designed to evaluate the performance improvement achieved by each module. 1. VIO: Only camera and IMU data were used for autonomous localization based on the PL-VINS framework [43,44], without introducing any UWB constraints. For consistent comparison, the estimated trajectories were transformed into the total station coordinate frame. 2. UWB: Only UWB ranging measurements were used for positioning, and two-dimensional locations were estimated via a robust Kalman filter. 3. UWB–VIO: The VIO and UWB results were independently transformed into a common coordinate frame and then fused using the Robust ESKF. 4. Proposed Method: The method proposed in this paper integrates joint initialization with multi-anchor directional consistency constraints and an improved Robust ESKF fusion algorithm.

**Figure 6:**
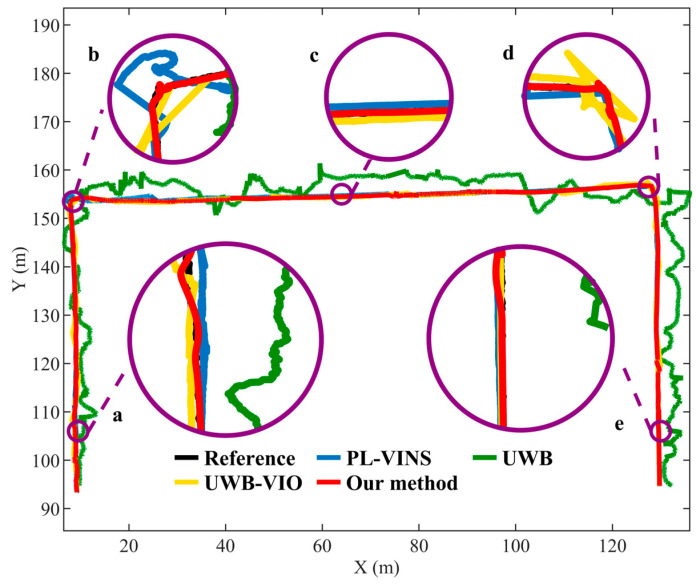
Trajectory Comparison Diagram. (a) Forward 100 m section; (b) First turning section; (c) Midpoint of the trajectory; (d) Second turning section; (e) End section of the trajectory.

To ensure fairness, all experiments were conducted along identical trajectories and under identical environmental conditions, and each configuration was repeated four times. The average positioning error metrics were then computed statistically. The ground-truth trajectory was obtained from high-precision total station tracking for reference calibration. To further validate the positioning accuracy and robustness of the proposed approach in complex indoor environments, trajectory estimation results from different algorithms were compared along identical motion paths. The trajectory tracked by the total station served as the reference benchmark, and the comparative trajectories from a representative experiment are illustrated in Figure 6 for visualization.

As shown in region (a) of Figure 6, the VIO system exhibits a noticeable drift after moving approximately 20 m forward, with its trajectory displaying a significant translational deviation from the ground-truth path during the return phase. This phenomenon mainly arises from the scale unobservability of VIO and the cumulative amplification of IMU integration errors over extended operation. In particular, within sections of the corridor characterized by sparse textures and pronounced illumination variations—such as region (c)—the number of matched visual features decreases substantially, resulting in discontinuities and jitter in the estimated trajectory.

The VIO attitude estimation becomes unstable in turning regions, such as locations (b) and (d) in Figure 6, owing to abrupt changes in motion direction and inadequate illumination. This instability further amplifies estimation errors. In contrast, the UWB system benefits from richer geometric constraints in these regions, maintaining higher positioning stability. In contrast, UWB positioning benefits from richer geometric constraints in these areas. The wider angular distribution of anchor points provides a higher-quality network geometry compared with straight sections, thereby reducing positioning errors. However, in other regions where the UWB anchors are approximately collinear, geometric degeneration becomes significant, weakening the vertical constraint capability. As a result, the trajectory exhibits noticeable fluctuations and drift, particularly in the central part of the corridor (region c in Figure 6). In this area, UWB positioning struggles to continuously and accurately capture the robot’s true motion trajectory.

The traditional UWB–VIO fusion algorithm alleviates the aforementioned issues to some extent, producing a trajectory that more closely aligns with the actual path and exhibits a noticeable reduction in translational deviation. However, due to inconsistencies in scale and yaw estimation during coordinate transformation, it remains challenging to fully unify observations across different coordinate frames during fusion. Consequently, rotational and stretching distortions still occur in the turning regions (b) and (d). Moreover, due to multipath interference and synchronization errors between coordinate systems, the fused trajectory exhibits localized instability in regions (c) and (e). Overall, although the fusion strategy improves the global consistency of the trajectory to some extent, its stability and adaptability still require further refinement.

In contrast, the proposed improved Robust ESKF fusion positioning method with UWB–VIO initialization exhibits excellent stability and continuity throughout the entire trajectory. The estimated trajectory aligns almost perfectly with the reference path, showing no noticeable drift or oscillation across regions (a–e). These results indicate that the proposed approach effectively suppresses coordinate system synchronization errors and scale drift, while maintaining high-precision global consistency even under challenging conditions such as low illumination and multipath interference.

The proposed method outperforms the comparison algorithms in terms of trajectory continuity, global consistency, and positioning accuracy. In particular, under long-range operations and multipath interference conditions, the proposed direction-consistent initialization combined with the improved Robust ESKF fusion framework enhances both system accuracy and robustness. By introducing a directional consistency constraint during initialization, the framework effectively mitigates scale drift and heading bias in subsequent fusion optimization. During filtering updates, the improved residual-weighted robust mechanism dynamically suppresses abnormal observations, ensuring stable state estimation over long trajectories in complex and dynamic environments.

Experimental results demonstrate that the proposed method effectively suppresses trajectory misalignment and attitude discontinuities observed in conventional VIO and loosely coupled UWB–VIO systems, while achieving smooth and high-precision trajectory estimation under challenging conditions such as UWB signal occlusion and weak visual features.

It should be noted that illumination robustness is not the primary focus of this study. The proposed system mainly aims to improve initialization consistency and suppress long-term drift through the UWB–VIO joint initialization strategy and the improved Robust ESKF fusion framework. Nevertheless, since parts of the experimental environment contain relatively low-light regions, the reported results inherently reflect system performance under mixed lighting conditions.

**Figure 7:**
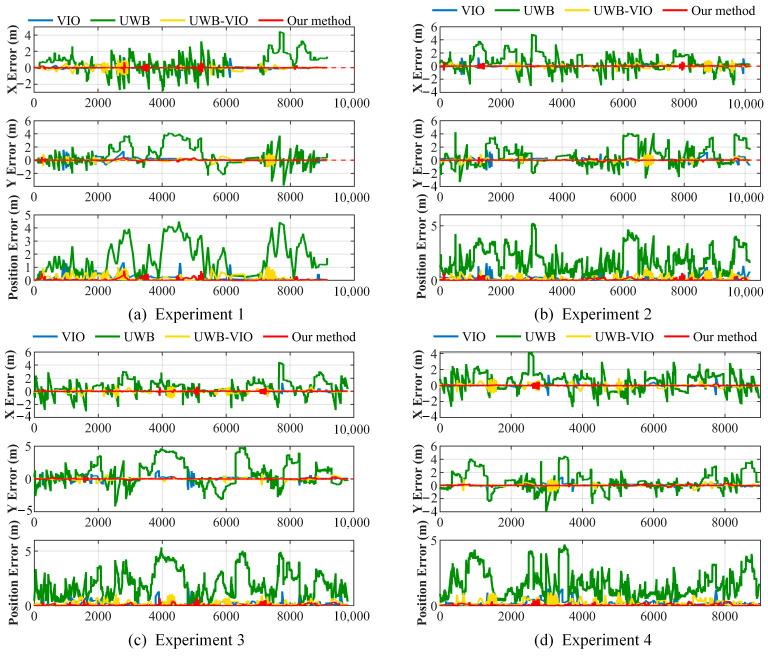
Positioning Error Curves of Four Repeated Experiments.

To quantitatively evaluate the positioning performance, the planar position error is adopted as the evaluation metric. The position error, as illustrated in Figure 7, is defined as the Euclidean distance between the estimated position and the reference ground truth in the horizontal plane. Each experimental configuration was repeated four times. The positioning error curves of the individual trials are presented to illustrate the consistency of the experimental results.

As shown in the error curves in Figure 7, the overall error level of the VIO system remains relatively low. However, short-term fluctuations still occur in regions with varying illumination or sparse textures, mainly due to unstable visual feature matching and accumulated IMU integration errors. Once sufficient visual features are reacquired, the error rapidly decreases, exhibiting the typical error recovery characteristics of visual–inertial systems. In the middle section of the corridor, where local illumination is dim and visual features are sparse, the number of matched features decreases, leading to transient fluctuations in the VIO error curve. In contrast, UWB positioning in this segment is affected by the nearly collinear geometric configuration of anchor points, resulting in reduced positioning accuracy. The corresponding error amplitude is larger and exhibits more severe fluctuations, with peaks exceeding 4 m, indicating that UWB localization is significantly influenced by geometric degradation and multipath interference in narrow corridor environments. Particularly in regions where anchor points are almost collinear, weakened vertical constraints lead to distinct peaks and irregular jumps in the error curve. The proposed method effectively compensates for these errors under geometrically degenerate conditions by jointly optimizing UWB and VIO, maintaining a continuous, stable trajectory with high positioning accuracy in such challenging areas.

For the UWB–VIO system, the overall error is significantly lower than that of single-sensor approaches, indicating that the complementary characteristics of the two sensors enhance global positioning constraints. However, at turning points (Region 3 in Figure 5), poor illumination and abrupt changes in motion direction reduce the number of visual feature matches in VIO, resulting in pronounced fluctuations in the error curve. The conventional UWB–VIO fusion method is susceptible to coordinate transformation errors and inconsistencies in heading estimation, leading to local oscillations in the error curve. In addition, trajectory misalignment often occurs during the initialization phase, causing a rapid increase in error at the early stage and occasional minor oscillations during the fusion process. In contrast, the proposed method introduces directional consistency constraints that effectively mitigate the effects of scale drift and heading bias on subsequent fusion optimization. Moreover, incorporating an improved residual-weighted robust mechanism into the filtering phase adaptively attenuates the impact of abnormal ranging measurements, enabling the system to maintain a smooth, stable error evolution even under varying illumination and multipath interference conditions.

Traditional algorithms exhibit varying degrees of error fluctuation across different scenarios. In contrast, the proposed UWB–VIO initialization-based improved Robust ESKF fusion positioning method demonstrates the most stable error evolution throughout the entire operation. The error curve shows low-amplitude oscillations, indicating that the algorithm maintains stable convergence even under multi-source uncertainties. Owing to the unified VIO–UWB coordinate system established during initialization and the improved residual-weighted robust mechanism introduced in the filtering stage, the system achieves better consistency and robustness. It can adaptively attenuate the influence of abnormal ranging measurements and effectively suppress errors caused by multipath interference. Even under challenging conditions such as uneven illumination and UWB signal occlusion, the proposed method maintains smooth trajectories and stable error behavior, fully validating its superior accuracy and robustness in complex indoor environments.

As shown in the error statistics in Table 1, which are computed as the average over four repeated experiments, the four methods exhibit significant differences in positioning accuracy and stability. The VIO system, which relies solely on visual and inertial measurements, maintains high short-range accuracy, with a positional root-mean-square error (RMSE) of 0.293 m. However, the maximum error reaches 1.588 m, indicating that scale drift and accumulated inertial errors gradually amplify with increasing travel distance, resulting in poor long-term stability. Although the UWB positioning system provides global constraints, it is susceptible to spatial geometric limitations in corridor environments and multipath interference.

Consequently, its positional RMSE increases to 2.192 m, with a maximum error of 4.486 m, showing the largest fluctuation range and evident instability caused by geometric degradation. After integrating UWB and VIO information, both the overall accuracy and trajectory consistency of the system are notably improved. The positional RMSE decreases to 0.279 m, and the maximum error reduces to 1.038 m—approximately 34.6% lower than that of VIO—demonstrating the effectiveness of UWB in mitigating VIO drift. However, due to inconsistencies in scale and yaw estimation between the two coordinate systems, the fusion results still exhibit localized drift during certain time intervals. In contrast, the proposed method achieves the best performance across all metrics. Its positional RMSE is only 0.105 m—representing a further reduction of approximately 64.3% compared to conventional UWB–VIO fusion—while the maximum error remains below 0.740 m. These results indicate excellent error convergence and stability during long-range operation in complex environments. The improved Robust ESKF–based UWB-VIO fusion framework effectively mitigates the effects of UWB ranging anomalies and VIO feature loss on overall accuracy, thereby enhancing positioning robustness and global consistency. Overall, the proposed approach demonstrates significant error convergence and accuracy improvements in complex indoor environments, validating the effectiveness and engineering practicality of the algorithm.

To further demonstrate the advantages of the proposed algorithm, trajectory errors in the turning regions were statistically analyzed, as shown in Table 2. The error statistics from the turning areas indicate that the positioning performance of all algorithms differs noticeably from that observed in straight-line segments. Due to the more widely distributed anchor geometry at turning points, the UWB network exhibits a significantly improved geometric configuration compared with the near-collinear layout in narrow corridors. As a result, the geometric constraint capability of the UWB system is enhanced in these regions, reducing the positional RMSE from 2.192 m in straight sections to 0.816 m, demonstrating a clear improvement in accuracy. However, the system remains affected by multipath reflections and signal occlusions, leading to significant error fluctuations, with a maximum error of 2.884 m. Meanwhile, the VIO system experiences a substantial reduction in the number of matched visual features during turning maneuvers due to rapid changes in viewing angles and lighting variations. Consequently, its trajectory estimation exhibits more pronounced drift and jitter than during the straight-line phase, and the positional RMSE increases to 0.588 m, indicating degraded accuracy under these conditions. The traditional UWB–VIO fusion method achieves overall higher accuracy than standalone VIO in this scenario, reducing the positional RMSE to 0.558 m. Nevertheless, due to coordinate system misalignment and uneven fusion weight allocation, local trajectory offsets still occur in regions with abrupt angular changes, with the maximum error remaining as high as 1.038 m.

In contrast, the proposed method maintains high positioning accuracy and trajectory continuity even in turning regions. It achieves a positional RMSE of only 0.326 m, which is approximately 41.6% lower than that of the traditional UWB–VIO fusion algorithm, while keeping the maximum error within 0.7 m. This demonstrates the system’s strong robustness under conditions involving rapid directional changes and multipath interference. These results highlight that the synergistic integration of the direction-consistent initialization and the improved robust fusion mechanism effectively alleviates the adverse effects of sudden heading variations on attitude estimation. Moreover, the enhanced robust filtering mechanism further suppresses multipath-induced ranging anomalies, enabling the system to maintain stable, continuous trajectory estimation even in sharp-turn scenarios with significant directional variation.

### 5.3. Real-Time Performance Analysis

To evaluate the computational efficiency of the proposed framework, experiments were conducted on a laptop equipped with an Intel Core i5-1135G7 processor (4 cores, 2.40 GHz) and 16 GB RAM. The system runs under Ubuntu 20.04 without GPU acceleration. The IMU operates at 200 Hz, the monocular camera at 10 Hz, and the UWB module at 5 Hz. The fusion update frequency is set to 5 Hz.

The joint optimization of scale and yaw is performed only during the initialization stage within a limited sliding window. The average computation time for initialization is approximately 48 ms. Since initialization is executed only once at system startup, it does not affect long-term runtime performance. During continuous operation, the state estimation is implemented using an improved Robust ESKF. Owing to the filtering-based formulation and the linear complexity with respect to the state dimension, the computational overhead remains lightweight. The detailed runtime statistics, including processing latency and CPU utilization, are summarized in Table 3.

As shown in Table 3, the fusion stage based on the Error-State Kalman Filter exhibits an average processing latency of 6.3 ms per update, with a maximum observed latency of 9.8 ms. Given the 5 Hz update rate (200 ms period), the computational load occupies only a small fraction of the available processing time. The average CPU utilization during sustained operation remains below 28%, indicating sufficient computational margin for additional tasks. The average processing latency per fusion update is significantly smaller than the 200 ms update period, and the CPU usage remains moderate during sustained operation. These results confirm that the proposed framework maintains stable real-time performance with sufficient computational margin, demonstrating its suitability for practical robotic deployment.

### 5.4. Influence of UWB Anchor Deployment Geometry

The spatial configuration of UWB anchors directly affects system observability and the geometric dilution of precision (GDOP). When anchors are sparsely distributed or exhibit unfavorable spatial arrangements, the directional observability of range measurements may degrade, potentially affecting both positioning accuracy and initialization stability. Although the primary focus of this work is not anchor layout optimization, clarifying the influence of anchor geometry is essential for understanding the practical applicability and boundary conditions of the proposed framework.

**Figure 8:**
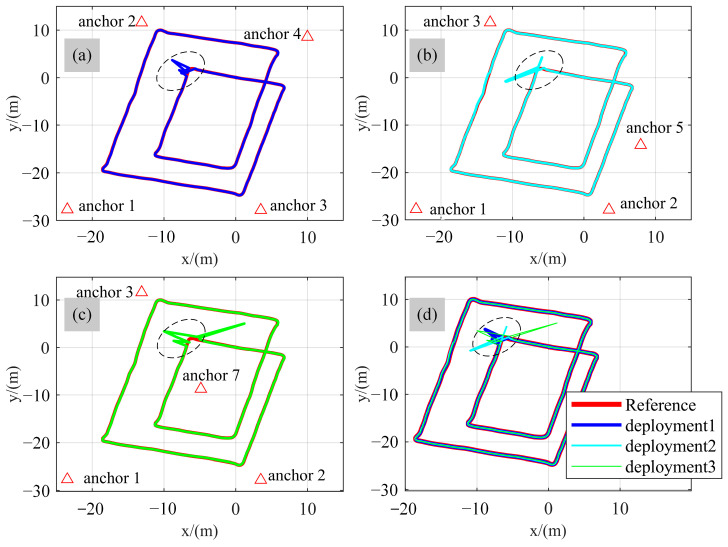
Positioning results of different UWB anchor deployment configurations. (**a**) Deployment 1; (**b**) Deployment 2; (**c**) Deployment 3; (**d**) Comparison of the three deployment configurations.

To systematically investigate this issue, an additional controlled experiment was conducted by varying the deployment configurations of the UWB anchors and evaluating their impact on localization performance. The positioning results under different configurations are illustrated in Figure 8, and the corresponding statistical errors are summarized in Table 4.

In this experiment, three representative anchor deployment configurations were designed. Each configuration employed four UWB base stations to ensure a consistent number of observations and identical experimental conditions. Environmental factors, hardware setup, and ranging parameters were kept unchanged across trials, so that the effect of geometry could be isolated. The overall measurement quality remained high, and no intentional disturbance was introduced.

In this analysis, localization is performed using the UWB-based positioning method proposed in this work. The VIO module is intentionally excluded so that the influence of anchor spatial distribution on the positioning performance can be investigated independently.

Specifically, Figure 8a presents Deployment 1, in which four base stations are arranged in a regular quadrilateral grid. This configuration provides relatively uniform spatial coverage and balanced angular distribution. Figure 8b illustrates Deployment 2, where the four base stations form an irregular quadrilateral layout, resulting in uneven angular separation. Figure 8c shows Deployment 3, consisting of one central base station and three surrounding base stations arranged in an approximately triangular configuration, leading to a more clustered spatial distribution.

The accuracy degradation observed in the dashed region can be mainly attributed to the spatial relationship between the robot trajectory and the anchor coverage area. In Deployment 2 and Deployment 3, part of the localization trajectory lies outside the region enclosed by the anchors. When the target moves beyond the anchor coverage area, the relative ranging geometry becomes less favorable, which weakens the effective geometric constraints provided by the measurements. Consequently, the geometric dilution of precision increases, leading to amplified localization errors in these regions [45].

From the statistical results in Table 4 and the trajectory comparisons in Figure 8a–c, it can be observed that when the number of anchors is identical and the ranging quality remains high, the three configurations achieve comparable overall positioning accuracy. The RMS and average errors along both horizontal axes remain within a similar magnitude. This indicates that under favorable observation conditions, anchor geometry has a limited influence on localization precision, provided that sufficient measurement redundancy exists.

However, a different behavior emerges when localized degradation in ranging quality occurs. As indicated by the dashed-line regions in Figure 8, certain time intervals exhibit reduced measurement reliability. Under these conditions, Deployment 2 and Deployment 3 show noticeably increased error fluctuations and larger maximum deviations. In contrast, Deployment 1 maintains comparatively smaller positioning errors and demonstrates improved stability. This phenomenon suggests that irregular or partially clustered anchor layouts are more sensitive to measurement degradation, as their geometric constraints are less uniformly distributed. When some range observations become unreliable, the remaining constraints may not sufficiently preserve directional observability, resulting in amplified positioning errors.

Among the three configurations, the regular quadrilateral arrangement in Figure 8a consistently achieves smaller maximum errors and better robustness under degraded observation conditions. The relatively symmetric spatial distribution enhances geometric conditioning, thereby mitigating the impact of partial measurement deterioration. These results demonstrate that anchor deployment geometry becomes a critical factor when observation quality is compromised, even if its influence appears limited under ideal ranging conditions.

Overall, this supplementary analysis indicates that while anchor geometry may not significantly affect localization accuracy in well-conditioned scenarios with high-quality measurements, it plays an important role in determining robustness under non-ideal conditions. The findings provide practical insight into deployment considerations for UWB-assisted systems and help contextualize the stability behavior observed in the subsequent system-level experiments.

### 5.5. Discussion

The experimental validation confirms that the proposed UWB–VIO fusion framework achieves improved trajectory continuity, global consistency, and long-term stability compared with conventional VIO and loosely coupled fusion approaches. The system-level evaluations verify the effectiveness of the proposed initialization and filtering strategies, while the supplementary analysis of anchor deployment geometry further clarifies the conditions under which ranging observability may influence overall performance.

A primary contributor to this improvement is the direction-consistent joint initialization strategy. By jointly estimating scale and heading alignment between the UWB and VIO coordinate systems, the method avoids the error accumulation that may arise from sequential or loosely constrained alignment procedures. The introduction of sliding-window-based trajectory direction smoothing further stabilizes the initialization stage. Instead of relying on instantaneous measurements, the method integrates short-term motion information to suppress the influence of transient noise, temporary geometric degeneration, and localized ranging fluctuations. This temporal consistency enhances the reliability of the initial state estimate and establishes a well-conditioned starting point for subsequent recursive filtering.

The second key component is the residual-weighted Robust ESKF employed during online fusion. Unlike conventional filtering schemes that treat UWB measurements uniformly, the proposed approach dynamically adjusts residual weights according to measurement consistency. This adaptive mechanism reduces the impact of abnormal ranging observations caused by multipath propagation, partial signal occlusion, or environmental interference. Consequently, the estimator maintains stable state evolution over extended trajectories even when measurement quality varies. The robustness advantage becomes particularly relevant under suboptimal anchor geometries or localized ranging degradation, where uniform weighting strategies are more prone to instability.

The narrow-corridor environment selected for evaluation intentionally imposes constrained anchor placement and increased susceptibility to multipath effects. Successful validation under such conditions indicates that the framework is capable of operating reliably in geometrically restrictive scenarios. In more open indoor environments, where anchor deployment can be optimized and spatial diversity is enhanced, comparable or improved localization performance is anticipated due to improved measurement conditioning.

Despite the demonstrated robustness, several limitations merit consideration. Severe UWB signal blockage, highly degenerate anchor configurations, or insufficient spatial diversity may reduce effective ranging observability and compromise absolute positioning accuracy. Under such conditions, the fusion framework gradually increases reliance on VIO propagation, which may introduce accumulated drift over long trajectories. Similarly, persistent visual degradation—such as texture-deficient regions, rapid illumination variations, or pronounced motion blur—can reduce local motion estimation accuracy. If both sensing modalities experience sustained degradation simultaneously, overall performance may decline. Furthermore, the current implementation primarily addresses planar motion scenarios and does not explicitly incorporate full three-dimensional observability for multi-floor environments.

Nevertheless, the framework is designed to degrade gracefully rather than fail abruptly. Through adaptive residual modulation and probabilistic state estimation, filter stability is preserved under partial sensor degradation. Compared with purely learning-based fusion approaches, the model-driven architecture adopted in this work does not require prior training data, maintains algorithmic interpretability, and ensures real-time computational efficiency. These characteristics support practical deployment on mobile robotic platforms operating in heterogeneous indoor environments.

Future research will investigate systematic anchor configuration optimization, enhanced three-dimensional observability modeling, and the integration of data-driven components to further improve robustness under extreme environmental conditions.

## 6. Conclusions

This paper addresses the challenges of scale inconsistency and heading errors in UWB–VIO fusion positioning. A novel direction-consistency-constrained UWB–VIO initialization method and an improved Robust ESKF–based fusion framework are proposed to achieve stable, high-precision positioning using UWB, visual, and inertial sensors in complex environments, such as narrow corridors and low-light conditions. In the initialization stage, a new multi-anchor direction consistency constraint model is introduced for joint optimization, effectively resolving the scale and heading unification problem and providing a reliable initial state for subsequent fusion. During the fusion stage, an enhanced residual-weighted Robust ESKF strategy is employed to adaptively suppress multipath and NLOS ranging errors, thereby significantly improving positioning accuracy and global consistency. Experimental results demonstrate that the proposed method maintains trajectory continuity and estimation stability even in environments with weak visual features and severe multipath interference, validating its robustness and practical applicability in complex indoor scenarios.

Although the proposed method demonstrates excellent performance in terms of accuracy and robustness, there is still room for further improvement. Future work will focus on extending the algorithm to complex three-dimensional environments such as multi-story buildings and multi-level warehouses, aiming to refine the spatial constraint model and enhance the system’s adaptability and positioning accuracy in fully three-dimensional scenarios.

## Figures and Tables

**Table 1:** Error Statistics Table.

Methods	X RMSE (m)	Y RMSE (m)	Position RMSE (m)	Position MAE (m)	Max Error (m)	Min Error (m)
VIO	0.137	0.258	0.293	0.183	1.588	0.014
UWB	1.365	1.715	2.192	1.884	4.486	0.188
UWB–VIO	0.209	0.186	0.279	0.196	1.038	0.017
Our method	0.072	0.077	0.105	0.057	0.740	0.015

**Table 2:** Error Statistics for Turning Areas.

Methods	X RMSE (m)	Y RMSE (m)	Position RMSE (m)	Position MAE (m)	Max Error (m)	Min Error (m)
VIO	0.371	0.456	0.588	0.443	1.588	0.017
UWB	0.525	0.596	0.816	0.659	2.884	0.188
UWB–VIO	0.321	0.459	0.558	0.482	1.038	0.164
Our method	0.221	0.240	0.326	0.291	0.700	0.015

**Table 3:** Runtime Performance of the Proposed Method.

Metric	Value
Processor	Intel Core i5-1135G7 (4 cores, 2.40 GHz)
Memory	16 GB RAM
Fusion update rate	5 Hz
Avg. processing latency	6.3 ms
Max. processing latency	9.8 ms
Initialization time	48 ms
CPU usage	28%

**Table 4:** Statistical of positioning errors for different base station layouts.

Deployment Configurations	RMS/m		Average/m		Max/m	
	x	y	x	y	x	y
Deployment 1	0.096	0.103	0.031	0.025	2.224	2.256
Deployment 2	0.159	0.221	0.037	0.036	2.773	4.919
Deployment 3	0.116	0.239	0.031	0.034	3.188	7.569

## Data Availability

The data obtained in the study are available from the corresponding author upon reasonable request.
